# Overexpression of Cathepsin L is associated with chemoresistance and invasion of epithelial ovarian cancer

**DOI:** 10.18632/oncotarget.10276

**Published:** 2016-06-24

**Authors:** Hongying Sui, Caixia Shi, Zhipeng Yan, Mei Wu

**Affiliations:** ^1^ Department of Gynecological Oncology, Hunan cancer hospital, The Affiliated Cancer Hospital of Xiangya School of Medicine, Central South University, Hunan, China

**Keywords:** ovarian cancer, CTSL, chemoresistance, SKOV3/TAX cells, proliferation

## Abstract

Paclitaxel is recommended as a first-line chemotherapeutic agent against, ovarian cancer, however, the development of chemoresistance is a major obstacle in patients with aggressive ovarian cancer and results in recurrence after conventional therapy. The key molecule or mechanism associated with paclitaxel resistance in ovarian cancer still remains unclear. Cathepsin L (CTSL) is overexpressed in various cancers, however, the association between CTSL expression and paclitaxel resistance remains unclear. In the present study, we investigated the role of CTSL in paclitaxel-resistant SKOV3/TAX cells by CTSL silencing. Expression of CTSL was examined by immunohistochemistry and qRT-PCR in 58 clinical samples, and in SKOV3 cells and SKOV3/TAX cells. Effects of CTSL knockdown on ovarian cancer cell proliferation, apoptosis, migration, and invasion were also studied. The IHC and real-time PCR results showed that the difference of CTSL expression between ovarian cancer and the adjacent non-tumourous ovarian tissues was statistically significant. Western blot analysis showed that the CTSL was overexpressed in SKOV3/TAX cells and weakly detectable in paclitaxel-sensitive SKOV3 cells. Knocking-down of CTSL in ovarian cancer cells could decrease cell proliferation, migration, and invasion, and potentiate apoptosis induced by paclitaxel, suggesting CTSL may contribute to Paclitaxel resistance in ovarian cancer.

## INTRODUCTION

Ovarian cancer is the leading cause of death from gynecological malignancies worldwide [1–2]. The overall 5-year survival rate is particularly low for the advanced stages. Current management strategies include debulking surgery and adjuvant chemotherapy with a regimen of platinum and paclitaxel, which has a response rate of 80% for all patients and 40–60% for advanced-stage patients [3]. Although improvement in median survival has been observed in recent decades, the majority of patients eventually succumb to recurrent, progressive disease due to resistance to chemotherapy [4–5]. Chemoresistance as an obstacle in the management is associated with a lower response rate to chemotherapy and a poor prognosis of ovarian cancer. Therefore, it is urgent to understand the mechanism of metastasis and resistance to chemotherapy in ovarian cancer.

Cathepsin L (CTSL), a lysosomal cysteine protease, is a member of the papain-like family of cysteine proteinases [6]. Accumulating evidence suggests that CTSL plays a role in cell proliferation, differentiation, apoptosis, angiogenesis, inflammation and extracellular tissue remodeling [7–8]. Elevated levels of CTSL are reported to be associated with a variety of malignant tumor [9–10]. Recent work has demonstrated that CTSL may also regulate cancer cell resistance to chemotherapy. Targeting CTSL alters the behavior of drug-resistant cancer cells. CTSL has been found to be overexpressed in ovarian cancer [11], however, the role of CTSL in chemoresistance is currently unknown. We hypothesized that high CTSL would be associated with intrinsic clinical drug resistance, manifesting as decreased time to disease progression/recurrence in patients. In this study, we investigated the expression and function of CTSL in ovarian cancer. We also determined whether CTSL could regulate chemoresistance in ovarian cancer cells. Our study revealed that inhibition of CTSL could enhance the chemosensitivity of ovarian cancer cells. The results of this study may clarify the role of CTSL in ovarian cancer and elucidate CTSL is a potential target for the treatment of patients with ovarian cancer.

## RESULTS

### CTSL expression was upregulated in human ovarian cancer tissues

**Figure 1:**
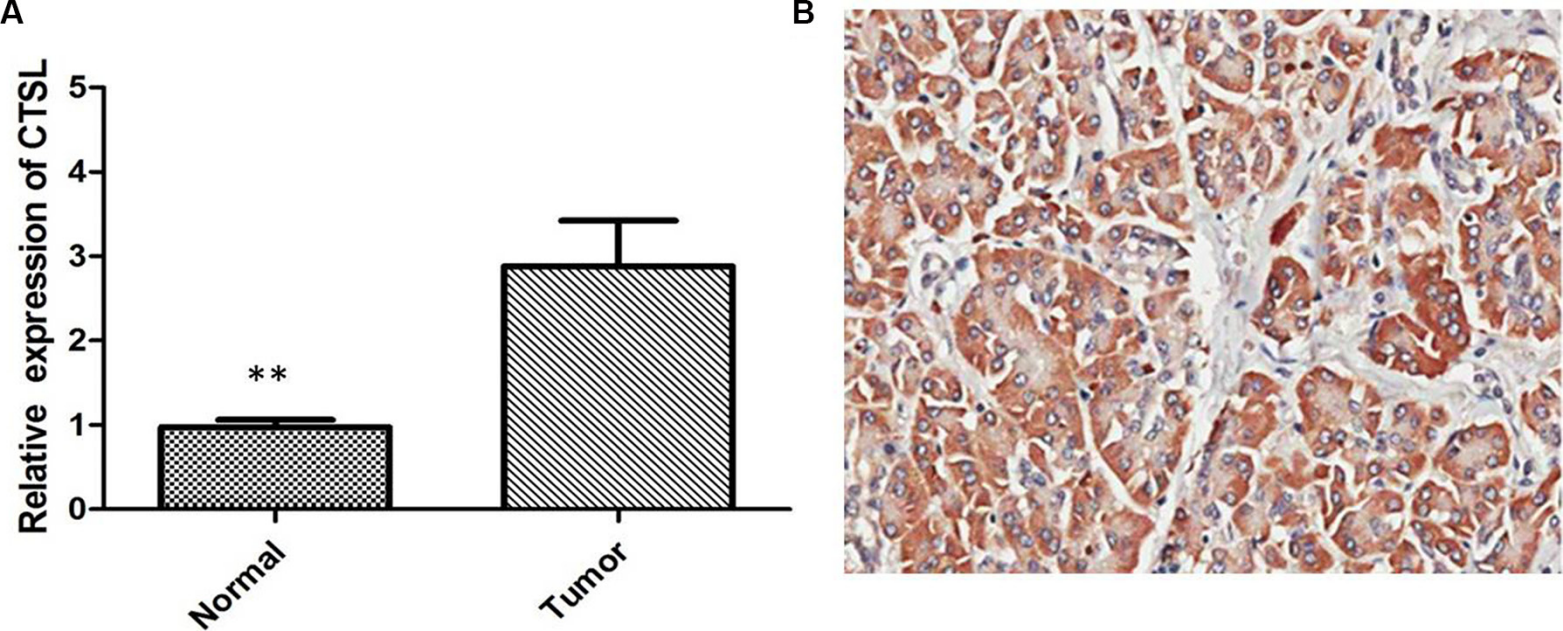
(**A**) Quantitative real-time PCR showing expression level of CTSL mRNA in ovarian cancer tissues (**indicates *p* < 0.01). (**B**) IHC showing high expression of CTSL in ovarian cancer tissues.

To determine the expression levels of CTSL in tumor tissues from human ovarian cancer patients, we assessed 58 pairs of ovarian cancer specimens and adjacent normal tissues by qRT-PCR. We found that the mRNA levels of CTSL were significantly higher in ovarian cancer tissues in comparison with adjacent normal tissues (Figure 1A, *P* < 0.01). We then examined the CTSL expression in clinical ovarian cancer specimens by IHC staining. The results showed that CTSL expression in ovarian cancer specimens was significantly upregulated compare with that in the adjacent non-tumoral tissue. High expression of CTSL was observed in 70.69% (41/58) of ovarian cancer specimens when compared with adjacent non-neoplastic tissues (32.76%, 19/58), the difference of CTSL expression was statistically significant (Figure 1B, *P* < 0.001), suggesting that CTSL may play an oncogenic role in ovarian cancer.

### CTSL expression was upregulated in paclitaxel-resistant ovarian cancer cell line

**Figure 2:**
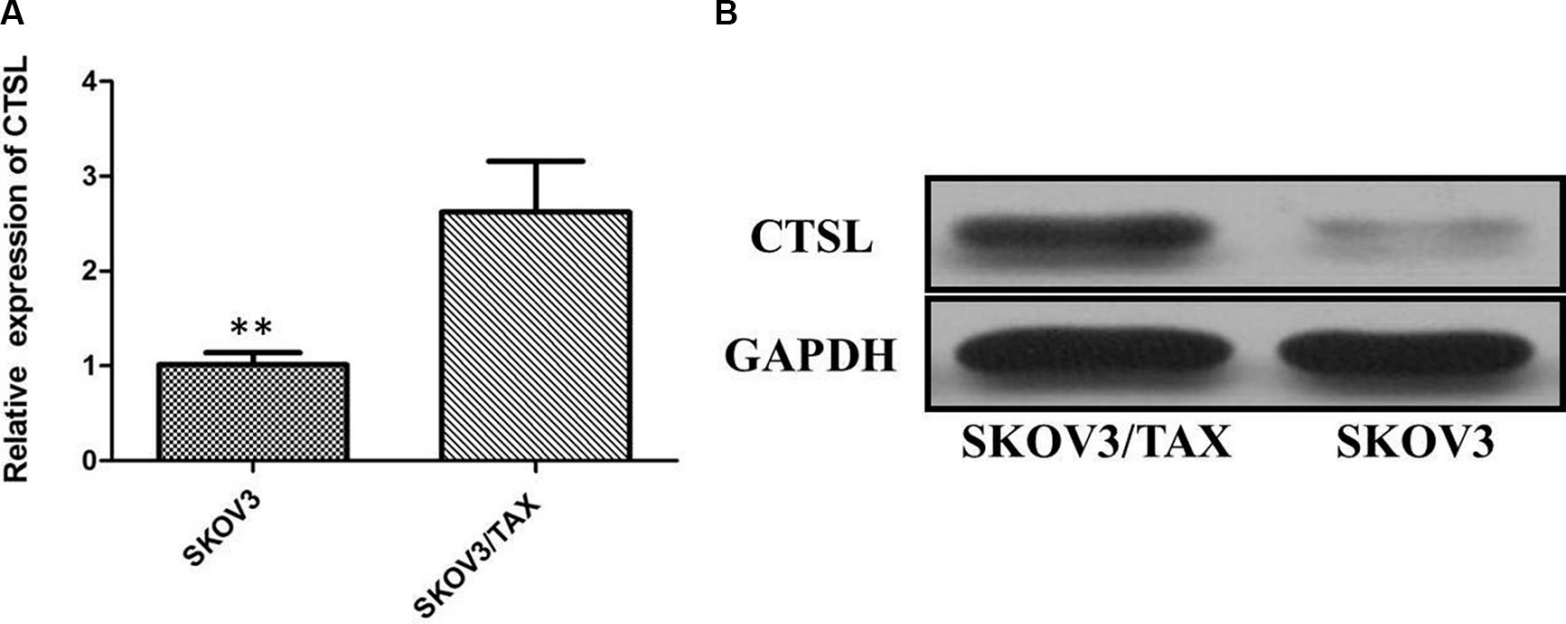
(**A**) Quantitative real-time PCR showing expression level of CTSL mRNA in SKOV3 and SKOV3/TAX cell lines (**indicates *p* < 0.01). (**B**) Western blots showing expression of SOX2 protein in SKOV3 and SKOV3/TAX cell lines.

**Figure 3:**
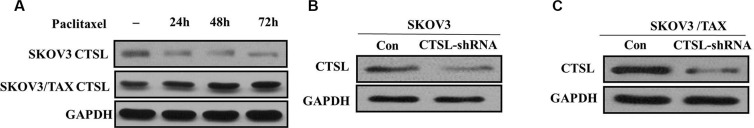
(**A**) The effect of paclitaxel treatment on the expression of CTSL in SKOV3 and SKOV3/TAX cell lines. (**B**) Western blots showing knocking-down of CTSL in SKOV3 cells. (**C**) Western blots showing knocking-down of CTSL in SKOV3/TAX cells;

To test the effect of CTSL level on modulation of chemosensitivity, SKOV3 and SKOV3/TAX cells were used to detect the expression of CTSL by qRT-PCR and Western blot assays. Our results have showed that the expression of CTSL was significantly increased in the paclitaxel-resistant cell line (SKOV3/TAX cells) compared with SKOV3 cells (Figure 2A). By Western blot analysis, we confirmed that the CTSL protein was overexpressed in paclitaxel-resistant cells, whereas its expression was weakly detectable in paclitaxel-sensitive cells (Figure 2B). In view of this, SKOV3 and SKOV3/TAX cells were used to study the effect of paclitaxel treatment on the expression of CTSL. Cells were treated with paclitaxel (100 nM) and harvested at various time points 24, 48 and 72 h. Intriguingly, immunoblotting showed CTSL expression to be decreased in SKOV-3 after the treatment of paclitaxel. However, CTSL expression remained relatively constant at high levels in SKOV3/TAX upon paclitaxel treatment (Figure 3A).

### CTSL knockdown inhibited proliferation of ovarian cancer cells

**Figure 4:**
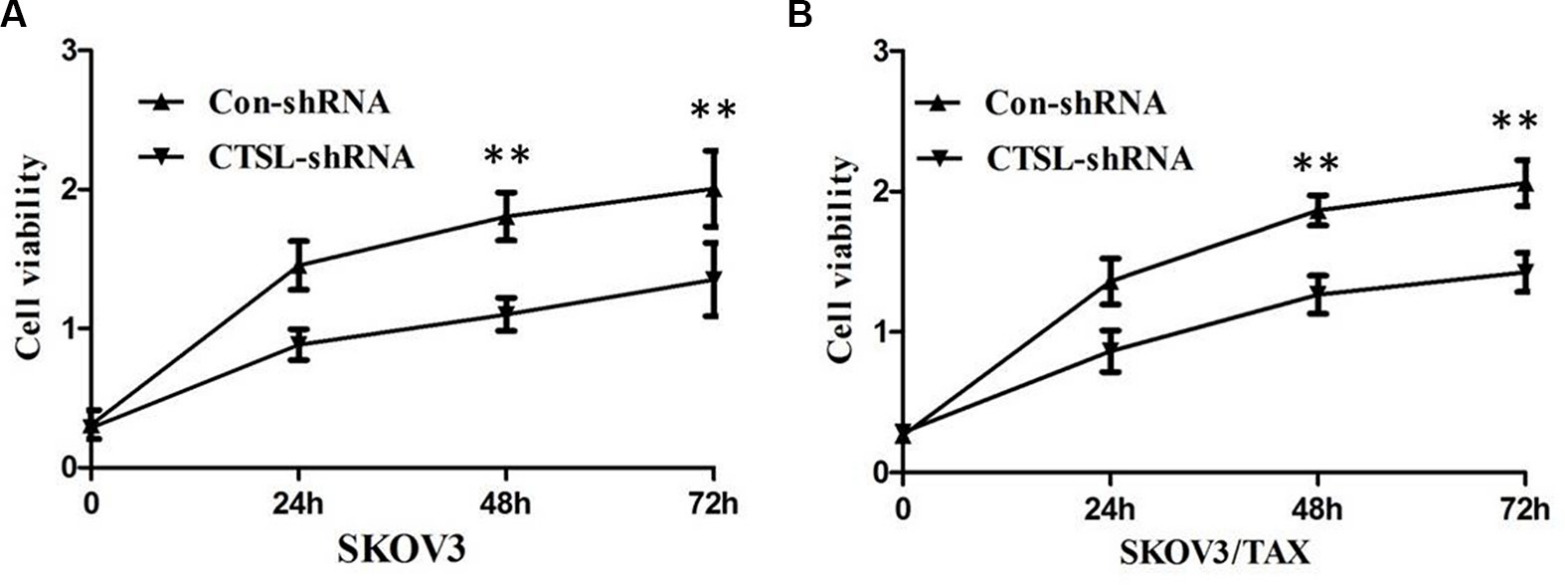
(**A**) MTT assay showing knocking-down of CTSL markedly suppressed the ability of proliferation of SKOV3 cells. (**B**) MTT assay showing knocking-down of CTSL markedly suppressed the ability of proliferation of SKOV3/TAX cells.

To further investigate the effect of CTSL in the proliferation ability of ovarian cancer cells, we established stable SKOV3 and SKOV3/TAX cell line with down-regulation of CTSL by shRNA sequences against CTSL (SKOV3/TAX-CTSL-shRNA). As shown in Figure 3B and 3C, CTSL shRNA transfection resulted in down-regulation of CTSL protein expression compared with control shRNA. We next studied the impact of CTSL silencing on ovarian cancer cell proliferation. The results of MTT assay showed that knockdown of CTSL in SKOV3 and SKOV3/TAX cells decreased cell proliferation (Figure 4A and 4B), suggesting that CTSL might be involved in the development of ovarian cancer.

### CTSL silencing promoted apoptosis induced by paclitaxel treatment in the resistant cell line SKOV3/TAX

**Figure 5:**
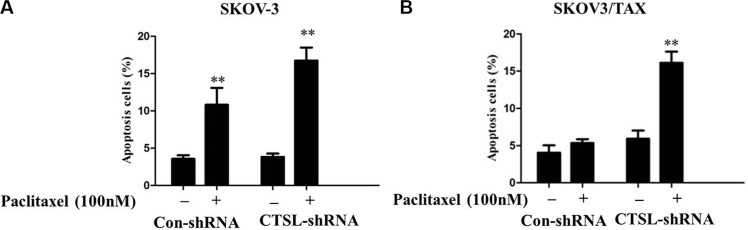
(**A**) Paclitaxel induced signicantly potentiated apoptosis in SKOV-3 cells transfected with either CTSL shRNA or control CTSL (**indicates *p* < 0.01). (**B**) CTSL silencing signicantly potentiated apoptosis induced by paclitaxel in SKOV3/TAX with CTSL knockdown compared with control shRNA (**indicates *p* < 0.01).

To clarify the possible mechanisms involved in CTSL knockdown sensitizing ovarian cancer cells to paclitaxel, annexin V assay of apoptosis was performed. To this end, SKOV3 and SKOV3/TAX cells were transfected with control and CTSL shRNA and cultured in the presence or absence of paclitaxel treatment (100 nM) for 48 h. The results showed that paclitaxel induced significantly potentiated apoptosis in SKOV-3 cells transfected with either CTSL or control. Interestingly, we found that CTSL silencing signicantly potentiated apoptosis induced by paclitaxel in SKOV3/TAX with CTSL knockdown compared to SKOV3/TAX transfected with control shRNA, suggesting CTSL contributes to paclitaxel resistance in ovarian cancer cells and that CTSL silencing can enhance paclitaxel mediated cell apoptosis (Figure 5A and 5B). Depletion of CTSL in the sensitive SKOV-3 cells has no additive effect to paclitaxel treatment. This is likely due to the fact that paclitaxel functions through down-regulating CTSL expression.

### Inhibition of CTSL suppressed the ability of cell migration and invasion of ovarian cancer cells

**Figure 6:**
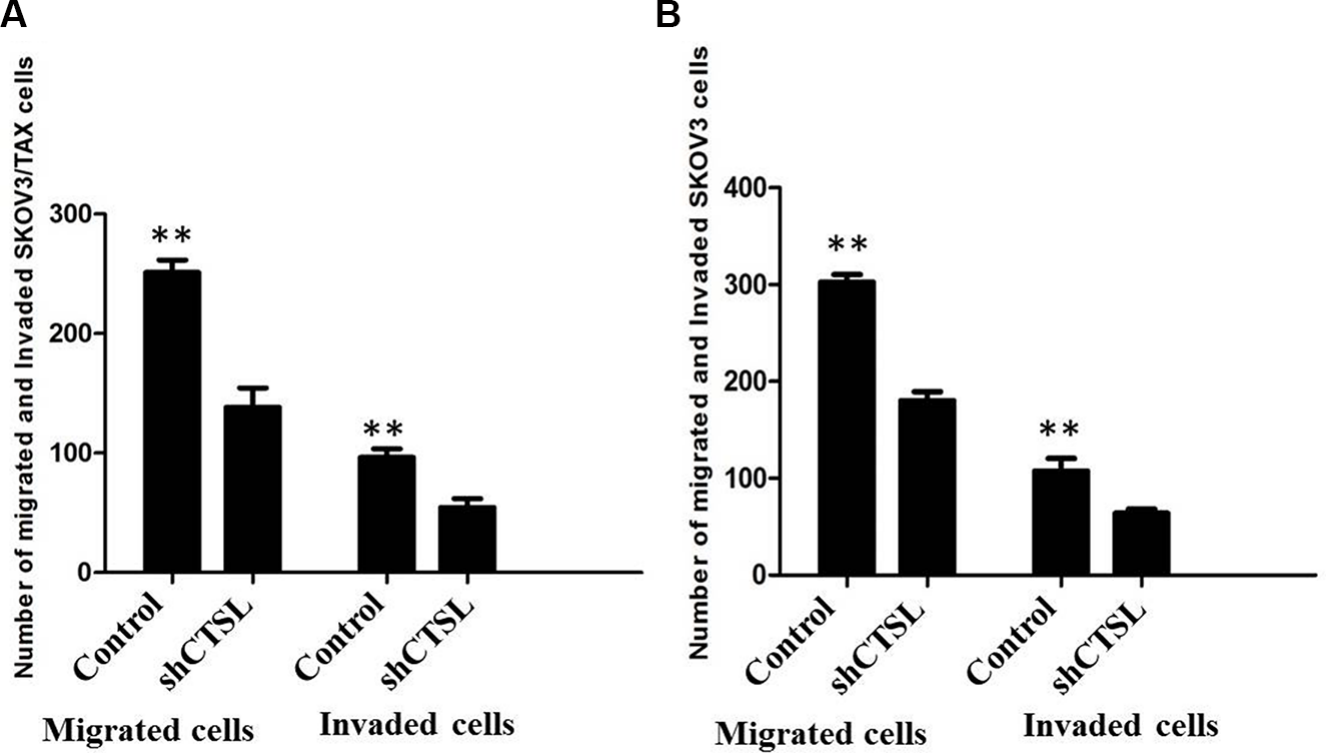
(**A**) Transwell assay showing knocking-down of CTSL markedly suppressed the ability of migration and invasion of SKOV3 cells. (**B**) MTT assay showing knocking-down of CTSL markedly suppressed the ability of migration and invasion of SKOV3/TAX cells.

We next studied the impact of CTSL silencing on ovarian cancer cell migration and invasion. The results of transwell assay showed that knockdown of CTSL in SKOV3 and SKOV3/TAX cells inhibited cell migration and invasion by comparing the number of cells invaded through the matrigel in shCTSL cells with those in control cells (Figure 6A and 6B).

## DISCUSSION

Ovarian cancer is the second most common gynecological malignancy and a major contributor to death from cancer in women [12]. While the initial response to first-line therapy (cytoreductive surgery and combined platinum-paclitaxel chemotherapy) is usually effective, most cancers recur, are chemotherapy-resistant, and result in the death of the patient [13]. An understanding of the molecular mechanisms underlying chemoresistance in ovarian cancer patients might aid the design of new treatment strategies. In our study, ovarian cancer cells resistant to paclitaxel showed upregulation of CTSL. CTSL, a lysosomal endopeptidase expressed in most eukaryotic cells, is a member of the papain-like family of cysteine proteinases, which is reported to be associated with cancer tumorigenesis, proliferation and migration [14]. CTSL has been proven to be upregulated in a variety of malignancies, and the level of CTSL expression is associated with the degree of malignancy [15–16].

In the current study, we firstly investigated the expression level of CTSL in the large sample size of 58 cases of ovarian cancer, and results showed that the expression level of CTSL mRNA was significantly increased in ovarian cancer tissues compared with normal paired tissues. IHC staining showed overexpression of CTSL was observed in 70.69% (41/58) of ovarian cancer specimens when compared with adjacent non-neoplastic tissues (32.76%, 19/58), the difference of CTSL expression was statistically significant. In addition, we noticed that the expression of CTSL was significantly increased in the paclitaxel-resistant cell line (SKOV3/TAX cells) compared with SKOV3 cells, suggesting that CTSL may play an oncogenic role in ovarian cancer.

To extend our clinical studies and investigate its biological function, we examined the effect of CTSL on the progression of ovarian cancer in SKOV3 and SKOV3/TAX cell model. We found that knockdown of CTSL expression by shRNA significantly reduced the proliferation rate of SKOV3 and SKOV3/TAX cells compared with the control vector, indicating that depletion of endogenous CTSL could attenuate the proliferation of ovarian cancer cells *in vitro*. Furthermore, CTSL silencing significantly potentiated apoptosis induced by paclitaxel in SKOV3/TAX compared with control shRNA, suggesting CTSL contributes to paclitaxel resistance in ovarian cancer cells and that CTSL silencing can enhance paclitaxel-mediated cell apoptosis. The rationale for the functional contributions of CTSL is based on its ability to target multiple effectors involved in these biological processes.

Accumulating evidence suggests may contribute its proteolytic action to the migration and invasion of tumor cells [17–18]. When the extracellular activity of CTSL is increased, the cell-cell adhesion is reduced and degradation of the ECM is increased [19]. In addition, CTSL also may switch the melanoma cell phenotype from non-metastatic to highly metastatic and increased tumor invasion and migration. We investigated the effect of CTSL knockdown on tumor invasion and migration. We demonstrated that CTSL is a regulator of invasion and migration of SKOV3 and SKOV3/TAX cells. Thus, CTSL is a novel target for reducing tumor progression.

In summary, these findings suggest that CTSL functions as a carcinogenic factor in human ovarian cancer tissues. Overexpression of CTSL was found in ovarian cancer tissues and cell lines. CTSL knockdown can enhance sensitivity of ovarian cancer cells to paclitaxel. Thus, CTSL may be a novel biological marker and potential therapeutic target for the treatment of ovarian cancer.

## MATERIALS AND METHODS

### Patients and specimens

Paraffin embedded tissue blocks were resected from the 58 patients with ovarian cancers admitted in the Department of Gynecological Oncology, Hunan cancer hospital, The Affiliated Cancer Hospital of Xiangya School of Medicine, Central South University between 2010 and 2014. All the specimens were confirmed as epithelial ovarian cancer by pathologists. The normal paired tissues were taken from the distal resection margins (more than 5 cm). None of the patients were treated with chemotherapy, immunotherapy or radiotherapy prior to specimen collection. The complete clinical pathologic characteristics were obtained including age and clinical stage of the patient, and differentiation of tumor cells. Besides, freshly frozen tissue samples were also available from these patients. Samples were snap-frozen in liquid nitrogen immediately after surgery and stored at −80°C until the analysis. This study was approved by the local institutional review board, and informed consent papers were obtained from all of the patients.

### Cell lines

SKOV3 cells (human ovarian adenocarcinoma cell line) and SKOV3/TAX cells (paclitaxel-resistant human ovarian adenocarcinoma cell line) were purchased from the Cell Bank of the Chinese Academy of

Sciences Institute (Shanghai, China). SKOV3 and SKOV3/TAX were cultured in RPMI1640 (Sigma) supplemented with 10% foetal bovine serum (FBS) and 100 units/ml penicillin-streptomycin (Invitrogen). All cell lines were maintained at 37°C in humidified incubator with 5% CO_2_.

### Immunohistochemistry (IHC)

Formalin-fixed, paraffin-embedded tissue blocks were cut into 4-μm sections and subjected to immunohistochemistry with rabbit polyclonal anti-CTSL antibody (1:50; Santa Cruz Biotechnology). IHC staining was performed according to previously published methods with minor modification [21]. IHC was carried out using the streptavidin peroxidase-conjugated method. Negative controls were prepared by substituting PBS substituting for primary antibody. All the immunoreactions were separately evaluated by two senior pathologists. For evaluating the expression of CTSL in ovarian cancer tissues, extent, intensity, and pattern of nuclear expression were assessed in each spot. All tissue sections were analyzed and scored independently by three experienced pathologists. A score was calculated by multiplying the intensity (negative scored as 0, mild scored as 1, moderate scored as 2 and strong scored as 3) by percentage of stained cells (0, < 5%; 1, 5–25%; 2, 26–50%; 3, 51–75%; and 4, 76–100%). Scores of multiplication were graded as follows: −, 0; +, 1–3; ++, 4–8; +++, 9–12. In addition, for statistical analysis, the − and 1+ cases were pooled into the low-expression group, and the 2+ and 3+ cases were pooled into the high-expression group.

### The qRT-PCR analysis

Total RNA was extracted using Trizol reagent (Invitrogen) according to the manufacturer's instructions. cDNA was synthesized from 1 mg of total RNA by use of the SuperScript III First-Strand Synthesis System (Invitrogen). Realtime PCR was carried out using CFX96 Real-Time System (BIORAD). SYBR green 26 master mixture (Invitrogen) was used in a total volume of 10 ml. The primer sequences were as follows: CTSL sense 5′- CTGGTGGTTGGCTACGGATT-3′, antisense 5′-CTCCGGTCTTTGGCCATCTT-3′, GAPDH sense 5′-TGTTGCCATCAATGACCCCTT-3′, antisense 5′-CTC CACGACGTACTCAGCG-3′, GAPDH was used as an internal control. All reactions were run in triplicate in three independent experiments.

### Western blotting analysis

Cell samples were solubilized in SDS lysis buffer, and the protein concentrations were detected by the BCA protein assay kit (PIERCE, Rockford, IL). Equal amounts of protein samples (30 mg/lane) were separated by electrophoresis through 9.0% resolving SDS–polyacrylamide gel, and then transferred to PVDF membranes (Amersham Pharmacia Biotech Inc in Piscataway, NJ). Block the non-specific binding sites by immersing the membrane into 5% non-fat milk in TBST solution for 1 hr, and then incubate the membrane with a primary monoclonal antibody to CTSL (1:1000Santa Cruz Biotechnology) for 2 hr at room temperature (RT). After washing 3 times in with TBST (TBS + 0.5% Tween-20), the membranes were incubated with a secondary antibody (diluted 1:1000 in TBS-T) for 1 hr at RT. The membranes were washed and proteins were detected by enhanced chemiluminescence system (Amersham Pharmacia Biotech) following manufacturer's instructions. Anti-GAPDH mouse monoclonal antibody was used to confirm equal loading of lysates (1:1000; Santa Cruz Biotechnology). Image J software was used to analyze the gray value.

### Vector construction and transfection

The pcDNA3.0 vector was used to generate pcDNA-CTSL. The CTSL shRNA Plasmid was purchased from Santa Cruz Biotechnology (Cat. No: sc-29939-SH). Vector transfection was performed according to the instructions, and cells were transfected with CTSL shRNA Plasmid or empty vector to knockdown the expression of CTSL.

### Cell viability assay

Cell viability was determined using the 3-(4,5)- dimethylthiahiazo(−z-y1)-3,5-di-phenytetrazoliumromide (MTT, Beyotime, Jiangsu, China) assay. Briefly, 2.5×103 cells/well were seeded to the wells of a 96-well plate and allowed to adhere. At different time points, 20 μL of MTT solution was added to each well of the plate, and the plates were incubated for 4 h. Then, liquid was removed from the plate and 150 μL of DMSO was added to the wells, the mixture was agitated for 10 minutes, and the OD was measured at 490 nm.

### Annexin V assay of apoptosis

At 48 h of siRNA transfection, the cells were exposed to indicated dose of paclitaxel. Then, cells were harvested and washed with ice-cold phosphate-buffered saline twice, suspended in annexin v-binding buffer and the indicated amount of propidium iodide and annexinV-FITC (BD Pharmingen, San Diego, CA, USA) was added. After incubation for 20 min at room temperature in the dark, fluorescence was measured on a flow cytometer.

### Cell invasion/migration assay

For the Transwell assay, Boyden chambers (8-μm pore size) were coated with 200-μl Matrigel at 200 μg/ml and incubated overnight. Cells (5 × 10^4^) that were seeded in medium without serum were plated in the upper chamber, and medium containing 10% FBS was added to the lower chamber as a chemoattractant. After incubation for 24 h at 37°C, the cells that migrated to the underside of the membrane were fixed in 4% formaldehyde and stained with crystal violet dye, and enumerated under a microscope. Three invasion chambers were used per condition. The values obtained were calculated by averaging the total number of cells from three filters.

### Statistical analysis

Data analyses were performed using SPSS statistical package 15.0 (SPSS Inc, USA). Results were presented as means of three independent experiments. Comparisons of percentage of viable cells and number of apoptotic cells among groups were performed using the two-tailed Student's *t*-test. Statistical significance was taken at the *P* < 0.05 level.
